# A Multi-Center, Real-World Study of Chidamide for Patients With Relapsed or Refractory Peripheral T-Cell Lymphomas in China

**DOI:** 10.3389/fonc.2021.750323

**Published:** 2021-11-04

**Authors:** Weiping Liu, Donglu Zhao, Ting Liu, Ting Niu, Yongping Song, Wei Xu, Jie Jin, Qingqing Cai, Huiqiang Huang, Zhiming Li, Ming Hou, Huilai Zhang, Jianfeng Zhou, Jianda Hu, Jianzhen Shen, Yuankai Shi, Yu Yang, Liling Zhang, Weili Zhao, Kaiyang Ding, Lugui Qiu, Huo Tan, Zhihui Zhang, Lihong Liu, Jinghua Wang, Bing Xu, Hui Zhou, Guangxun Gao, Hongwei Xue, Ou Bai, Ru Feng, Xiaobing Huang, Haiyan Yang, Xiaojing Yan, Qingshu Zeng, Peng Liu, Wenyu Li, Min Mao, Hang Su, Xin Wang, Jingyan Xu, Daobin Zhou, Hongyu Zhang, Jun Ma, Zhixiang Shen, Jun Zhu

**Affiliations:** ^1^ Key Laboratory of Carcinogenesis and Translational Research (Ministry of Education), Department of Lymphoma, Peking University Cancer Hospital and Institute, Beijing, China; ^2^ Department of Hematology and Oncology, Harbin Institute of Hematology and Oncology, Harbin, China; ^3^ Department of Hematology, West China Hospital Sichuan University, Chengdu, China; ^4^ Department of Hematology, Henan Cancer Hospital, Zhengzhou, China; ^5^ Department of Hematology, Jiangsu Province Hospital, Nanjing, China; ^6^ Department of Hematology, The First Affiliated Hospital of Zhejiang University, Hangzhou, China; ^7^ Department of Medical Oncology, Sun Yat-sen University Cancer Center, Guangzhou, China; ^8^ Department of Hematology, Qilu Hospital Shandong University, Jinan, China; ^9^ Department of Lymphoma, Tianjin Medical University Cancer Institute and Hospital, Tianjin, China; ^10^ Department of Hematology, Tongji Hospital Huazhong University of Science and Technology, Wuhan, China; ^11^ Department of Hematology, The Affiliated Union Hospital of Fujian Medical University, Fuzhou, China; ^12^ Department of Medical Oncology, The Cancer Institute and Hospital Chinese Academy of Medical Sciences, Beijing, China; ^13^ Department of Lymphoma, Fujian Cancer Hospital, Fuzhou, China; ^14^ Department of Medical Oncology, Union Hospital Affiliated to Tongji Medical College of Huazhong University of Science and Technology, Wuhan, China; ^15^ Department of Hematology, Shanghai Rui Jin Hospital, Shanghai, China; ^16^ Department of Hematology, Anhui Provincial Cancer Hospital, Hefei, China; ^17^ Department of Hematology, The Hematology Institute and Hospital Chinese Academy of Medical Sciences, Tianjin, China; ^18^ Department of Hematology, The First Affiliated Hospital Guangzhou Medical University, Guangzhou, China; ^19^ Department of Medical Oncology, Sichuan Cancer Hospital, Chengdu, China; ^20^ Department of Hematology, The Fourth Hospital of Hebei Medical University, Shijiazhuang, China; ^21^ Department of Medical Oncology, Nanjing General Hospital of Nanjing Military Command, Nanjing, China; ^22^ Department of Hematology, The First Affiliated Hospital of Xiamen University, Xiamen, China; ^23^ Department of Lymphoma, Hunan Cancer Hospital, Changsha, China; ^24^ Department of Hematology, Xijing Hospital of Airforce Medical University, Xi’an, China; ^25^ Department of Lymphoma, The Affiliated Hospital of Qingdao University, Qingdao, China; ^26^ Department of Hematology, The First Bethune Hospital of Jilin University, Changchun, China; ^27^ Department of Hematology, Nanfang Hospital of Southern Medical University, Guangzhou, China; ^28^ Department of Hematology, Sichuan Academy of Medical Sciences and Sichuan Provincial People’s Hospital, Chengdu, China; ^29^ Department of Lymphoma, Zhejiang Cancer Hospital, Hangzhou, China; ^30^ Department of Hematology, The First Hospital of China Medical University, Shenyang, China; ^31^ Department of Hematology, The First Affiliated Hospital of Anhui Medical University, Anhui, China; ^32^ Department of Hematology, Zhongshan Hospital Fudan University, Shanghai, China; ^33^ Department of Lymphoma, Guangdong Provincial People’s Hospital, Guangzhou, China; ^34^ Department of Hematology, People’s Hospital of Xinjiang Uygur Autonomous Region, Urumchi, China; ^35^ Department of Lymphoma, The Fifth Medical Center of the People's Liberation Army (PLA) General Hospital, Beijing, China; ^36^ Department of Hematology, Shandong First Medical University Affiliated Provincial Hospital, Jinan, China; ^37^ Department of Hematology, Nanjing Drum Tower Hospital of Nanjing University Medical School, Nanjing, China; ^38^ Department of Hematology, Peking Union Medical College Hospital, Beijing, China; ^39^ Department of Hematology, Peking University Shenzhen Hospital, Shenzhen, China

**Keywords:** lymphoma, T-cell, peripheral, histone deacetylase inhibitors, efficiency, safety, survival

## Abstract

Chidamide has demonstrated significant clinical benefits for patients with relapsed/refractory (R/R) PTCL in previous studies. This multi-center observational study was aimed to evaluate the objective response rate (ORR), overall survival (OS), and safety of chidamide. From February 2015 to December 2017, 548 patients with R/R PTCL from 186 research centers in China were included in the study. Among the 261 patients treated with chidamide monotherapy, ORR was 58.6% and 55 patients (21.1%) achieved complete response (CR). Among the 287 patients receiving chidamide-containing combination therapies, ORR was 73.2% and 73 patients (25.4%) achieved CR. The median OS of all patients was 15.1 months. The median OS of patients receiving chidamide monotherapy and combination therapies was 433 and 463 days, respectively. These results demonstrate a significant survival advantage of chidamide treatments as compared with international historical records. Common adverse effects (AEs) were hematological toxicities. Most AEs in both monotherapy and combined treatments were grade 1–2. No unanticipated AEs occurred. In conclusion, chidamide-based therapy led to a favorable efficacy and survival benefit for R/R PTCL. Future studies should explore the potential advantage of chidamide treatment combined with chemotherapy.

## Introduction

Peripheral T-cell lymphoma (PTCL) is a rare and heterogeneous group of clinically aggressive mature T- and natural killer (NK)-cell neoplasms associated with poor prognosis. Twenty-seven different types of PTCL are described in the 2016 revision of the World Health Organization classification of lymphoid neoplasms. PTCL represents 10–15% of non‐Hodgkin lymphomas (NHLs) in Western countries and accounts for about 25–30% of NHLs in China (1, 2). Moreover, the subtype distribution of PTCL is different between China and Western countries. The most common subtype of PTCL in China is extranodal NK/T-cell lymphoma (NKTCL), nasal type, followed by PTCL-not otherwise specified (PTCL-NOS), anaplastic large-cell lymphoma (ALCL), and angioimmunoblastic T-cell lymphoma (AITL) (2, 3).

For relapsed or refractory PTCL, conventional chemotherapy without intensification is usually associated with high treatment failure and disease relapse rates (3–5). Novel agents that target various pathways, such as histone deacetylase (HDAC) inhibitors, have been intensively studied and developed. Epigenetic therapies is also supported by identifying mutations of epigenetic genes in different PTCL subtypes, including *TET2*, *IDH2*, *RHOA*, *DNMT3A*, *CD28*, and *FYN* (6–10). Chidamide, a novel benzamide class of HDAC inhibitors, has been demonstrated to block the catalytic pocket of class I HDACs and selectively inhibit the activity of HDAC1, 2, 3, and 10 (11–17). For relapsed/refractory (R/R) PTCL, chidamide led to an overall response rate (ORR) of 28% in a phase II study (18) and an ORR of 39% in a real-world study (19). This study was a single arm, open-label, retrospective, post-marketing observational study of chidamide. The primary objective was to evaluate the safety, efficacy, and survival benefit of chidamide-containing therapy for relapsed or refractory (R/R) PTCL.

## Methods

### Patients and Study Design

The current study’s protocol was approved by the Institutional Review Board of all of the participating centers and was in accordance with the Declaration of Helsinki. Written informed consent was waived owing to the use of a deidentified data set.

From February 2015 to December 2017, patients with R/R PTCL from 186 research centers in China were enrolled in the study. The main inclusion criteria were as follows: PTCL subtypes being relapsed or refractory disease as defined by histologic pathology, and receiving chidamide-containing therapy with a duration more than six weeks. When monotherapy was chosen, a dose of 30 mg chidamide was orally administered twice weekly. When combined with other regimens, chidamide with a dose of 20–30 mg twice a week was given consecutively or according to physicians’ choices.

The response criteria was based on the Lugano classification recommendation for response assessment of Hodgkin lymphoma and non-Hodgkin lymphoma (20). ORR was defined as the proportion of patients achieving complete remission (CR) and partial response (PR). OS was calculated from the initiation of chidamide until death or the final follow-up (June 2018). Safety assessment was graded according to the Common Toxicity Criteria for Adverse Events scale, v4.03 (CTCAEv4.03).

### Statistics

Data analysis was conducted using IBM SPSS for Windows software (Version 25.0; IBM Corp). A chi-square test was used for comparison of categorical variables, and a *t* test was used for comparison of continuous variables. Kaplan-Meier method was employed for survival analysis. Multivariate analysis for OS was performed using the Cox proportional hazards model.

## Results

### Patient Characteristics

A total of 548 patients with R/R PTCL were enrolled in the study. The baseline characteristics of the patients are summarized in **Table 1**. The median age was 57 years (range, 18–89 years), with a male/female ratio of 1.6:1. More than one half of the patients received chidamide-containing combination treatments, in which a cytotoxic drug was predominant (**Supplement Table 1**).

**Table 1:** Baseline characteristics of 548 patients with relapsed or refractory PTCL.

Characteristic	Number of patients (%)
Total	548
Sex	
Male	341 (62.2)
Female	207 (37.8)
Age	
≤60 years	332 (60.6)
>60 years	216 (39.4)
ECOG PS	
0–1	336 (61.3)
2–4	212 (38.7)
Pathology type	
AITL	177 (32.3)
PTCL-NOS	220 (40.1)
ALCL	41 (7.5)
ALK-positive	12 (2.2)
ALK-negative	11 (2.0)
ALK-unknown	18 (3.3)
NKTCL	66 (12.0)
Others	44 (8.0)
IPI	
Low	124 (22.6)
Low-intermediate	173 (31.6)
High-intermediate	157 (28.6)
High	94 (17.2)
Treatment lines	
2^nd^ line	224 (40.9)
3^rd^ line	133 (24.3)
4^th^ line or beyond	64 (11.7)
Data missing	127 (23.2)
Stage	
I–II	66 (12.1)
III- IV	471 (85.9)
Data missing	11 (2.0)
B symptoms	
With B symptoms	169 (30.8)
Without B symptoms	102 (18.6)
Data missing	277 (50.5)

PTCL, peripheral T-cell lymphoma; ECOG, Eastern Cooperative Oncology Group; PS, performance status; AITL, angioimmunoblastic T-cell; PTCL-NOS, peripheral T-cell lymphoma, not otherwise specified; ALCL, anaplastic large-cell lymphoma; ALK, anaplastic lymphoma kinase; NKTCL, natural killer/T-cell lymphoma; IPI, International Prognostic Index.

### Efficacy

For the entire cohort, the ORR and CR rate were 66.2% and 23.4%, respectively. The best ORR was observed in AITL (75.1%), followed by ALCL (70.7%), PTCL-NOS (61.4%), and NKTCL (53.0%, **Table 2**). The CR rates varied from 20% to 30% according to different pathology, but was not statistically significant.

**Table 2:** Efficacy of chidamide-based treatment stratified by baseline characteristics.

	CR		ORR	
	N (%)	*P*	N (%)	*P*
Age		0.031		0.842
≤60	88 (26.5)		221 (66.6)	
> 60	40 (18.5)		142 (65.7)	
Gender		0.892		0.47
Male	79 (23.2)		222 (65.1)	
Female	49 (23.7)		141 (68.1)	
ECOG PS		0.177		0.004
0–1	85 (25.3)		238 (70.8)	
2–5	43 (20.3)		125 (59.0)	
Stage	124 (23.1)	0.197	356 (66.3)	0.426
I–II	16 (24.2)		48 (72.7)	
III- IV	53 (22.9)		131 (65.4)	
Pathology		0.55		0.006
AITL	53 (29.9)		133 (75.1)	
PTCL-NOS	44 (20.0)		135 (61.4)	
ALCL	10 (24.4)		29 (70.7)	
NKTCL	16 (24.2)		35 (53.0)	
Others	5 (11.4)		31 (70.5)	
IPI score		0.391		0.115
Low risk			87 (70.2)	
Low-intermediate risk	39 (22.5)		123 (71.1)	
High-intermediate risk	33 (21.0)		97 (61.8)	
High risk	20 (21.3)		56 (59.6)	
Treatment line	90 (21.4)	0.672	276 (65.6)	0.212
2^nd^ line	51 (22.8)		155 (69.2)	
3^rd^ line	25 (18.8)		80 (60.2)	
≥ 4^th^ line	4 (21.9)		41 (64.1)	

CR, complete response; ORR, overall response rate; ECOG, Eastern Cooperative Oncology Group; PS, performance status; AITL, angioimmunoblastic T-cell lymphoma; PTCL-NOS, peripheral T-cell lymphoma, not otherwise specified; ALCL, anaplastic large-cell lymphoma; NKTCL, natural killer/T-cell lymphoma, IPI, International Prognostic Index.

Chidamide-containing combination therapies exhibited a better ORR (73.2% vs. 58.6%, *P* < 0.001) as compared with chidamide monotherapy, but had similar CR (25.4% vs. 21.1%) rates. Among the 261 patients treated with chidamide monotherapy, 55 (21.1%) patients achieved CR, 98 (37.5%) achieved PR, and 80 (30.7%) achieved SD. Of the 287 patients receiving chidamide-containing combination therapies, 73 (25.4%) patients achieved CR, 137 (47.8%) achieved PR, and 49 (17.0%) achieved SD. The differences in either the CR rate or ORR between different combination regimens were not statistically significant.

### Safety

The most common adverse events (AEs) were neutropenia (46.7%) in patients treated with chidamide monotherapy, and fatigue (89.2%) in those treated with chidamide-containing combination therapies. Neutropenia was the most common grade 3-4 AE. The incidences and severity of AEs were significantly higher in patients receiving combination treatments than in those receiving the monotherapy (**Table 3**). There was no unanticipated AEs during the follow-up period.

**Table 3:** Adverse events.

	Monotherapy		Combination therapy	
	Grade 1–2	Grade 3–4	Grade 1–2	Grade 3–4
Neutropenia	81 (31.0)	41 (15.7)	80 (27.9)	106 (36.9)
Anemia	68 (26.1)	19 (7.3)	113 (39.4)	54 (18.8)
Thrombocytopenia	82 (31.4)	30 (11.5)	93 (32.4)	91 (31.7)
Fatigue	89 (34.1)	16 (6.1)	167 (58.2)	89 (31.0)
Fever	31 (11.9)	0 (0)	58 (20.2)	7 (2.4)
Nausea/vomiting	59 (22.6)	3 (1.1)	99 (34.5)	4 (1.4)
Diarrhea	35 (13.4)	2 (0.8)	44 (15.3)	3 (1.0)
Prolonged QTc period	6 (2.3)	1 (0.4)	8 (2.8)	0 (0)
Thromboembolism	2 (0.8)	0 (0)	14 (4.9)	0 (0)
Elevated ALT	16 (6.1)	4 (1.5)	40 (13.9)	2 (0.7)
Elevated AST	14 (5.4)	5 (1.9)	29 (10.1)	4 (1.4)
Elevated Creatinine	7 (2.7)	0 (0)	11 (3.8)	1 (0.3)
Proteinuria	8 (3.1)	0 (0)	13 (4.5)	0 (0)

ALT, alanine transaminase; AST, aspartate transaminase; QTc, QT interval corrected by heart rate.

### Survival

**Figure 1:**
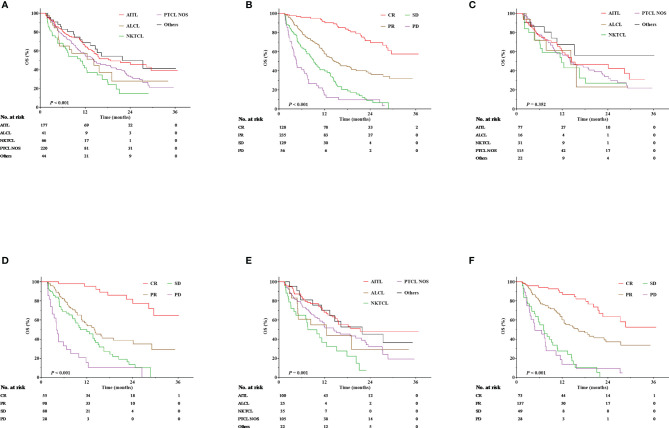
Overall survival (OS) according to pathological subtypes and treatment responses. **(A)** OS according to pathological subtypes for the entire cohort. **(B)** OS according to treatment responses for the entire cohort. **(C)** OS according to pathological subtypes for those treated with chidamide monotherapy. **(D)** OS according to treatment responses for those treated with chidamide monotherapy. **(E)** OS according to pathological subtypes for those treated with chidamide-containing combined therapies. **(F)** OS according to treatment responses for those treated with chidamide-containing combined therapies.

A total of 260 patients died during the follow-up period. The median OS was 15.1 months (range, 12.9–17.4 months), and the anticipated 1- and 2-year OS rates were 57.9% and 35.8%, respectively, for the entire cohort. In terms of pathological subtypes, the anticipated 1- and 2-year OS rates were 64.2% and 45.4%, respectively, for AITL; 50.7% and 27.7%, respectively, for ALCL; 41.8% and 14.5%, respectively, for NKTCL; 54.2% and 32.0%, respectively, for PTCL-NOS; and 65.4% and 41.4%, respectively, for other types (*P* < 0.001, **Figure 1A**). The survival benefit varied according to treatment responses, with an anticipated 1- and 2-year OS rate of 90.4% and 69.4%, 58.1% and 36.1%, 39.7% and 8.7%, and 12.2% and 6.5% for patients achieving CR, PR, SD, and progression disease (PD), respectively (*P* < 0.001, **Figure 1B**).

The median follow-up was 4.9 months. Among patients treated with chidamide monotherapy, the expected 1- and 2-year OS rates were 58.0% and 36.5%, respectively, for all patients; 58.8% and 42.5%, respectively, for those with AITL; 46.0% and 23.0%, respectively, for those with ALCL; 48.5% and 27.0%, respectively, for those with NKTCL; 56.4% and 31.8%, respectively, for those with PTCL-NOS; and 67.2% and 56.0%, respectively, for those with other types (*P* = 0.352, **Figure 1C**). In terms of treatment responses, the expected 1- and 2-year OS rates were 95.3% and 77.5%, 53.8% and 34.9%, 47.7% and 10.1%, and 10.3% and 0 for patients achieving CR, PR, SD, and PD, respectively (*P* < 0.001, **Figure 1D**).

Among patients receiving chidamide-containing combination therapies, the expected 1- and 2-year OS rates were 57.3% and 35.2%, respectively, for all patients; 68.3% and 47.8%, respectively, for those with AITL; 43.2% and 28.8%, respectively, for those with ALCL; 32.2% and 7.4%, respectively, for those with NKTCL; 51.8% and 32.0%, respectively, for those with PTCL NOS; and 64.5% and 45.2%, respectively, for those with other types (*P* = 0.001, **Figure 1E**). In terms of treatment responses, the expected 1- and 2-year OS rates were 86.7% and 63.4%, 60.2% and 37.1%, 27.5% and 0, and 13.9% and 4.6% for patients achieving CR, PR, SD, and PD, respectively (*P* < 0.001, **Figure 1F**).

## Discussion

The current large-scale, real-world study explored the safety, efficacy, and survival benefit of chidamide for R/R PTCL. Chidamide-containing therapy led to a satisfactory efficacy with a ORR of 73.2% and good tolerance without unanticipated AEs. Moreover, chidamide-containing therapy brought a survival advantage with a 2-year OS rate of 35.8%. Especially for those patients achieving CR, both chidamide monotherapy and combination therapy resulted in improved survival outcome with the 2-year OS of more than 60%.

Previous studies have shown that HDAC inhibitors have significant anticancer potential for R/R PTCL. In a phase II study involving 131 patients, romidepsin led to rapid response with a median time to objective response of 1.8 months, and resulted in an ORR of 25% and a CR rate of 15% (21). During the long-term follow-up period, the median DOR for all responders was 28 months, and 32% of patients achieving CR had a DOR of more than 24 months (22). In a real-world study, romidepsin resulted in an ORR of 33%, a CR rate of 12.5%, and a median DOR of 13.4 months (23). Similarly, a pivotal phase II study showed the ORR of belinostat led to an ORR of 25.8% with a CR rate of 10.8% (24). In the current study, the ORR of chidamide-containing therapy was 66.2% for the entire cohort. Notably, a relatively higher response rate was observed in AITL with an ORR of 75.1% and a CR rate of 29.9%. AITL is characterized by high frequencies of mutations in epigenetic modifiers in neoplastic T cells (9), which can partly explain the significant clinical benefits of chidamide. In addition, the efficacy of chidamide seemed to be higher than that of pralatrexate which led to an ORR of 29% with a CR rate of 11% for relapsed or refractory PTCL (25), but it was lower than that of Brentuximab vedotin which led to an ORR of 86% with a CR rate of 57% for ALCL (26).Therefore, future studies focusing on the impact of HDAC inhibitors on the survival benefit of specific subtypes are needed.

Survival expectations for patients with R/R PTCL treated with salvage chemotherapy is very poor. A retrospective study demonstrated that patients with first-time relapsed PTCL treated with chemotherapy only had a median OS of 6.5 months (27). In contrast, HDAC inhibitors showed a better survival advantage. Romidepsin resulted in a median DOR of 28 months and a median PFS of 29 months, of which a better survival benefit was observed in those who achieved CR for ≥ 12 months (22). In the current study, the overall median OS for all patients was 15.1 months, and the 2-year OS rate was 69.4% for patients achieving CR, suggesting a significantly improved long-term survival benefit of chidamide to patients with R/R PTCL.

Chidamide was generally well-tolerated in the current study. Most of the AEs were hematological toxicities of grades 1–2, including thrombocytopenia, neutropenia, and anemia. The incidence of AEs slightly increased in patients receiving chidamide-containing combination treatments, but all AEs were manageable. Transient prolongation of QT interval corrected by heart rate (QTc) period was observed, which was not associated with concurrent cardiac symptoms. Therefore, this study further confirmed the safety of chidamide both in monotherapy and along with other chemotherapies.

There was several limitations in the current study. First, the time to response was taken into account when the inclusion criteria was developed. The median time to objective response for romidepsin was 1.8 months (21), while chidamide led to a rapid response with 74% of all responses occurring within the first 6 weeks after treatment (18). Based on these reports, patients who received therapy with a duration more than six weeks were enrolled to explore the long-term survival benefit of chidamide in the current study. However, it resulted in a significant selection bias for the evaluation of efficacy, which led to a higher ORR (58.6%) than that reported in a previous real-world study (ORR was 51.2%) (19). Second, the optimal combined cytotoxic drugs were not determined due to the heterogeneous regimens during combined therapy, and data of salvage therapy after disease progression was not collected. Third, many baseline characteristics data including central pathology review, clinical manifestation, imaging examination methods for staging and response, and prognosis except international prognostic index was missing due to multicenter nature and enrollment, which made it difficult to select particular patient population who potentially benefitted from chidamide therapy.

In conclusion, the current large-scale study demonstrated that chidamide had a favorable efficacy and a tolerable safety profile for patients with R/R PTCL. In addition, the current study demonstrated the potential survival benefit of chidamide for patients with R/R PTCL when combined with chemotherapy.

## Data Availability Statement

The raw data supporting the conclusions of this article will be made available by the authors, without undue reservation.

## Ethics Statement

The studies involving human participants were reviewed and approved by Institutional Review Board of all of the participating centers. The ethics committee waived the requirement of written informed consent for participation.

## Author Contributions

WpL conceived and designed the study, analyzed the data, and drafted and revised the paper. DLZ prepared and analyzed the data. JuZ, JM, and ZS conceptualized and designed the study. All authors provided critical comments to the manuscript. All authors contributed to the article and approved the submitted version.

## Conflict of Interest

The authors declare that the research was conducted in the absence of any commercial or financial relationships that could be construed as a potential conflict of interest.

The reviewer JH declared a shared affiliation, with WZ and a past co-authorship with JJ, TL, LQ, WC, JH, and PL to the handling editor at the time of the review.

## Publisher’s Note

All claims expressed in this article are solely those of the authors and do not necessarily represent those of their affiliated organizations, or those of the publisher, the editors and the reviewers. Any product that may be evaluated in this article, or claim that may be made by its manufacturer, is not guaranteed or endorsed by the publisher.
