# Supplementary material for: A Multi-Center, Real-World Study of Chidamide for Patients With Relapsed or Refractory Peripheral T-Cell Lymphomas in China
Source: Front Oncol. 2021 Nov 4;11:750323. doi: 10.3389/fonc.2021.750323 (PMC8602952; doi:10.3389/fonc.2021.750323)
Supplement: Supplementary file 1 [file Table_1.docx]

Supplement Table 1. Distribution of regimens in 548 patients receiving chidamide-containing therapies

|  | Number (%) | Definition and agents |
| --- | --- | --- |
| Monotherapy | 261 (47.6) | Chidamide alone |
| Combination therapies | 287 (52.4) |  |
| With cytotoxic drugs | 258 (47.1) | Regimens containing cytotoxic drugs |
| CHOP | 52 (9.5) | Cyclophosphamide, doxorubicin, vincristine, and prednisone |
| EPOCH | 32 (5.8) | Etoposide, prednisone, vincristine, cyclophosphamide, and doxorubicin |
| ICE | 11 (2.0) | ifosfamide, carboplatin, and etoposide |
| DICE | 5 (0.9) | dexamethasone, etoposide, ifosfamide, and cisplatin |
| DHAP | 2 (0.4) | dexamethasone, high-dose cytarabine, and cisplatin |
| ESHAP | 1 (0.2) | etoposide, methylprednisolone, high-dose cytarabine, and cisplatin, |
| GDP | 26 (4.7) | gemcitabine, dexamethasone, and cisplatin |
| GemOx | 11 (2.0) | gemcitabine, oxaliplatin |
| PCT | 21 (3.8) | prednisone, cyclophosphamide, and thalidomide |
| PET | 8 (1.5) | prednisone, etoposide, and thalidomide |
| MPEC | 9 (1.6) | methotrexate, prednisone, etoposide and cyclophosphamide |
| P-GemOX | 4 (0.7) | pegaspargase, gemcitabine, oxaliplatin |
| Others | 76 (13.9) | Other regimens |
| With non-cytotoxic drugs | 29 (5.3) | non-cytotoxic drugs |
| Glucocorticoid | 8 (1.5) | prednisone |
| Thalidomide | 16 (2.9) | Thalidomide |
| Lenalidomide | 5 (0.9) | Lenalidomide |
